# The structure of anticorrelated networks in the human brain

**DOI:** 10.3389/fnetp.2022.946380

**Published:** 2022-11-16

**Authors:** Endika Martinez-Gutierrez, Antonio Jimenez-Marin, Sebastiano Stramaglia, Jesus M. Cortes

**Affiliations:** ^1^ Biocruces-Bizkaia Health Research Institute, Barakaldo, Spain; ^2^ Dipartamento Interateneo di Fisica, Universita Degli Studi di Bari Aldo Moro, INFN, Bari, Italy; ^3^ Biomedical Research Doctorate Program, University of the Basque Country, Leioa, Spain; ^4^ Department of Cell Biology and Histology, University of the Basque Country, Leioa, Spain; ^5^ IKERBASQUE Basque Foundation for Science, Bilbao, Spain

**Keywords:** anticorrelated networks, precuneus, cerebellum, default mode network, ageing, resting state, functional magnetic resonance imaging

## Abstract

During the performance of a specific task--or at rest--, the activity of different brain regions shares statistical dependencies that reflect functional connections. While these relationships have been studied intensely for positively correlated networks, considerably less attention has been paid to negatively correlated networks, a. k.a. anticorrelated networks (ACNs). Although the most celebrated of all ACNs is the default mode network (DMN), and has even been extensively studied in health and disease, for systematically all ACNs other than DMN, there is no comprehensive study yet. Here, we have addressed this issue by making use of three neuroimaging data sets: one of N = 192 healthy young adults to fully describe ACN, another of N = 40 subjects to compare ACN between two groups of young and old participants, and another of N = 1,000 subjects from the Human Connectome Project to evaluate the association between ACN and cognitive scores. We first provide a comprehensive description of the anatomical composition of all ACNs, each of which participated in distinct resting-state networks (RSNs). In terms of participation ranking, from highest to the lowest, the major anticorrelated brain areas are the precuneus, the anterior supramarginal gyrus and the central opercular cortex. Next, by evaluating a more detailed structure of ACN, we show it is possible to find significant differences in ACN between specific conditions, in particular, by comparing groups of young and old participants. Our main finding is that of increased anticorrelation for cerebellar interactions in older subjects. Finally, in the voxel-level association study with cognitive scores, we show that ACN has multiple clusters of significance, clusters that are different from those obtained from positive correlated networks, indicating a functional cognitive meaning of ACN. Overall, our results give special relevance to ACN and suggest their use to disentangle unknown alterations in certain conditions, as could occur in early-onset neurodegenerative diseases or in some psychiatric conditions.

## Introduction

It is well known that anatomical networks, such as those built from diffusion tensor imaging (DTI), introduce statistical dependencies in the dynamics of connected regions, increasing their functional connectivity (FC) (Friston, 2011; Diez et al., 2015). However, FC can also occur between regions with no direct anatomical connections (Beckmann et al., 2005; Buckner et al., 2013), due to the effects of common inputs or physiological elements, or the so-called indirect effects that refer to correlations between two regions that arise from neighboring regions (Friston, 2011; Alonso-Montes et al., 2015; Amor et al., 2015; Diez et al., 2015; Stramaglia et al., 2017; Fernandez-Iriondo et al., 2021; Safari et al., 2021). Functionally connected networks can be decomposed into positively correlated networks and negatively correlated networks, the latter widely known as anticorrelated networks (ACN). When derived from functional magnetic resonance imaging (MRI), some preprocessing steps might enhance the presence of ACN, like performing global signal regression (Murphy et al., 2009; Saad et al., 2012; Murphy et al., 2018), but accumulated evidence has shown that CAN could fulfil a physiological role independently on the details of the preprocessing (Fox et al., 2005; Fox et al., 2009; Uddin et al., 2009; Chai et al., 2012; Li et al., 2019). The most celebrated ACN is the default mode network (DMN), widely shown to be anticorrelated with multiple-task activation networks (Uddin et al., 2009; Chen et al., 2017). ACNs might also facilitate task-switching, as shown for the Dorsal Attention Network (Elton and Gao, 2014), Salience Network and the Executive Control Network when switching from resting state to task performance (Dosenbach et al., 2006; Chiong et al., 2013). Here, we extend these results by studying each resting state network (RSN) and its corresponding ACN, hypothesising that novel relationships can be found between two study groups by comparing the structure of the ACN, over and above the classic differences found when comparing them with positive correlated networks. To confirm our hypothesis, we use the condition of physiological aging and assess the differences between groups of old and young subjects in ACN. Finally, to assess the cognitive relevance of ACN, we study the association between cognitive performance and ACN, emphasizing some functional role of ACN in relation to cognitive performance.

## Materials and methods

### Participants

Data were selected from the 1,000 functional connectomes project and downloaded from the FCP Classic Data Table (available at http://fcon_1000.projects.nitrc.org/fcpClassic/FcpTable.html) (FCP, 2022). To identify and analyse ACN, we chose the *Beijing Eyes-Open Eyes-Closed II (Beijing EOEC2)* dataset (Chao-Gan, 2017), selecting N = 192 subjects with no history of neurological or psychiatric disorders (age range 18–26 years, mean 21.17, std. dev 1.83; males = 74). To analyse the differences in ACN produced by aging, we chose The Max Planck Institute Leipzig Mind-Brain-Body dataset–LEMON (Babayan et al., 2019; Mendes et al., 2019), selecting 20 young participants (range 20–25 years; 10 male, 10 female) and 20 old participants (range 70–80 years; 10 male, 10 female). To analyse the relevance of ACN in relation with cognitive performance, we used the Human Connectome Project (HCP) dataset (Connectome Coordination Facility, 2011), WU-Minn Consortium (Principal Investigators: David Van Essen and Kamil Ugurbil; 1U54MH091657) funded by the 16 NIH Institutes and Centers that support the NIH Blueprint for Neuroscience Research; and by the McDonnell Center for Systems Neuroscience at Washington University. In particular, raw images and cognitive scores were taken from N = 1,000 healthy adult subjects (ages range 22–37 years, mean = 28.68, std. dev = 3.69; males = 464).

### Cognitive measurements

To measure cognitive performance, we used the scores total and crystallized cognition adjusted by age (CogTotalComp_AgeAdj and CogEarlyComp_AgeAdj) from HCP, well-known for encompassing multiple cognitive functions from the NIH Toolbox for Assessment of Neurological and Behavioral Function (available at www.nihtoolbox.org) in a single score. The Total Cognitive Function Composite (CogTotalComp) was obtained by averaging the normalized scores of each of the Fluid (CogFluidComp) and Crystallized (CogCrystalComp) cognition tests. In particular, CogFluidComp is the combination of Flanker, Dimensional Change Card Sort, Picture Sequence Memory, List Sorting and Pattern Comparison; and CogCrystalComp is the combination of Picture Vocabulary and Reading Tests. The Early Childhood Composite (CogEarlyComp) was obtained by averaging the normalized scores Picture Vocabulary, Flanker, DCCS and Picture Sequence Memory. The Age-adjusted Scale (AgeAdj) was chosen within the age-appropriate band of the Toolbox Norming Sample.

### Brain imaging acquisition and analyses

#### Beijing dataset

The MRI data were acquired on a SIEMENS Trio 3-Tesla scanner at Beijing Normal University. Functional data were acquired using the following parameters: TR = 2s; TE = 30 ms; 33 axial slices with thickness/gap = 3/0.6 mm; volumes = 230; functional resolution was 3.125 × 3.125 × 3 mm with in-plane resolution of 64 × 64 voxels and FOV = 200 × 200 mm^2^. The T1-weighted sagittal three-dimensional magnetization-prepared rapid gradient echo (MPRAGE) sequence was acquired using the following imaging parameters: 128 slices; TR = 2,530 ms; TE = 3.39 ms; slice thickness = 1.33 mm; flip angle = 7°; inversion time = 1,100 ms; FOV = 256 × 256 mm^2^.

#### LEMON dataset

MRI raw and pre-processed images are available at (Index of/pub/misc/MPI-Leipzig_Mind-Brain-Body-LEMON, 2018) and although the full details of data acquisition are given at (Max Planck Institute, 2013; Mendes et al., 2019), we summarize here the main acquisition parameters. High-resolution structural images were acquired through a 3D MP2RAGE sequence (Marques et al., 2010) using the following parameters: voxel size = 1.0 mm isotropic, FOV = 256 × 240 × 176 mm, TR = 5,000 ms, TE = 2.92 ms, TI1 = 700 ms, TI2 = 2,500 ms, flip angle 1 = 4°, flip angle 2 = 5°, bandwidth = 240 Hz/Px, GRAPPA acceleration with iPAT factor 3 (32 reference lines), pre-scan normalization, duration = 8.22 min. Functional images were acquired through four rs-functional(f)MRI runs, all in an axial orientation using T2*-weighted gradient-echo echo planar imaging (GE-EPI) with multiband acceleration. The acquisition parameter for all four runs were: voxel size = 2.3 mm isotropic, FOV = 202 × 202 mm^2^, imaging matrix = 88 × 88, 64 slices with 2.3 mm thickness, TR = 1,400 ms, TE = 39.4 ms, flip angle = 69°, echo spacing = 0.67 ms, bandwidth = 1776 Hz/Px, partial Fourier 7/8, no pre-scan normalization, multiband acceleration factor = 4,657 volumes, duration = 15 min 30 s. During the resting-state scans, the participants were instructed to remain awake with their eyes open and to fix their vision on a crosshair.

#### HCP dataset

For each HCP subject, MRI acquisition was performed using a 3T Siemens Connectome Skyra with a 100 mT/m and 32-channel receive coils. High-resolution T1-weighted images were acquired with a 3D magnetization prepared rapid acquisition gradient echo (MPRAGE) and the following scanning parameters: TR = 2,400 ms, TE = 2.14 ms, voxel size = 0.7 × 0.7 × 0.7 mm^3^, slice thickness = 5.0 mm, flip-angle = 8º, FOV = 224 × 224 mm^2^ and acquisition-time = 7 min and 40 s. Resting state functional data was acquired through an EPI sequence with a duration of 14 min 33 s and the following parameters: 1,200 brain volumes, TR = 720 ms, TE = 33.1 ms, FOV = 208 × 180 mm^2^, flip-angle = 52º, voxel size = 2 × 2 × 2 mm^3^, matrix = 104 × 90, slice thickness = 2.0 mm, and 72 slices per volume.

### Image preprocessing

#### Beijing and LEMON datasets

Functional images of the two datasets were pre-processed using the Functional Connectivity (CONN v18b) toolbox (Whitfield-Gabrieli and Nieto-Castanon, 2012), applying the default pipeline for volume-based analyses that consists of functional realignment and unwarping, translation to a common reference of functional images, slice timing correction, artefact detection tool based identification of outlier scans with a 97% percentile threshold, functional segmentation and normalization, translation to a common reference of structural images, structural segmentation and normalization, and functional smoothing with a Gaussian kernel of full width at half maximum equivalent to 8 mm. De-noising included regressing out 5 white matter components and 5 components of the cerebral spinal fluid, 12 realignment time series, scrubbing, linear de-trending and applying a band-pass filter between 0.008 and 0.09 Hz. Global signal regression was not performed.

#### HCP dataset

The N = 1,000 functional images were pre-processed in a previous work (Fernandez-Iriondo et al., 2022) by correcting for gradient distortions and normalized to the standard MNI152 template of voxel size equal to 2 × 2 × 2 mm^3^ using the HCP pipelines *fMRIVolume* and *fMRISurface*. After image normalization, we eliminated nuisances with a procedure that combines a volume censoring strategy and motion-related time course regression, along with physiological signal regression. For this, the volumes were marked as censored when the frame displacement (FD) was greater than 0.2 or the root mean square derivative of the variance was greater than 0.75%, following previous recommendations (Power et al., 2013; Power et al., 2014; Parkes et al., 2018). In addition, the volume before the censored one and the two after they were also marked as censored. Next, the entire time series was divided into segments of 5 volumes in length, to finally eliminate all the segments that contained at least one contaminated volume, as well as the first segment. After that, nuisances were removed while simultaneously applying a bandpass filter between 0.01 and 0.08 Hz. Nuisance signals were the first five principal components of the CSF and WM signals; linear and quadratic trends; and the 24-parameter motion-related time series. Global signal regression was not performed. Finally, each filtered image was spatially smoothed with a 6 mm FWHM Gaussian kernel.

### Statistical analysis

We analysed eight different brain networks using seed-based correlation analysis (SBC). One seed was chosen within each of the networks available in the *networks* atlas incorporated in CONN (Whitfield-Gabrieli and Nieto-Castanon, 2012) and composing of 32 Regions of Interest (ROIs). In addition, a different CONN atlas was used for the anatomical description of significant clusters with 132 ROIs resulting from the combination of the FSL Harvard-Oxford atlas cortical and subcortical areas (Makris et al., 2006) and the AAL cerebellar areas (Tzourio-Mazoyer et al., 2002). After applying SBC, we built ACNs for each of the following RSNs: Default Mode Network (DMN), Fronto Parietal Network (FPN), Sensorimotor Network (SMN), Dorsal Attention Network (DAN), Visual Network (VN), Language Network (LN), Salience Network (SN), and Cerebellar Network (CN). The seeds used for SBC to generate each network were the medial posterior cingulate cortex (DMN), the left lateral prefrontal cortex (FPN), the left lateral sensorimotor (SMN), the left ipsilateral region (DAN), the medial visual (VN), the left inferior frontal gyrus (LN), the anterior cingulate cortex (SN), the cerebellum posterior region (CN), respectively.

For the first analysis of ACN anatomical description (using the Beijing dataset), a one-sample t-test was performed to build ACN. Voxel-level multiple correction was performed using p-FDR-corrected (p-False Discovery Rate) with a significance threshold of 10^^−14^ and peak p-FWE-corrected (p-Family Wise Error) with a threshold equal to 10^^−14^. For the second analysis of group ACN comparison between old and young participants (using the LEMON dataset), we performed two-sample t-test. Voxel-level correction was performed with p-FDR-corrected<0.05 and cluster size p-FDR-corrected<0.05. For the association of ACN with cognitive scores (using the HCP dataset), we fitted generalized linear model (GLM) independently for each voxel. Voxel-level significance was assessed by p-uncorrected<0.05 and cluster size p-FDR-corrected<0.05.

## Results

**FIGURE 1:**
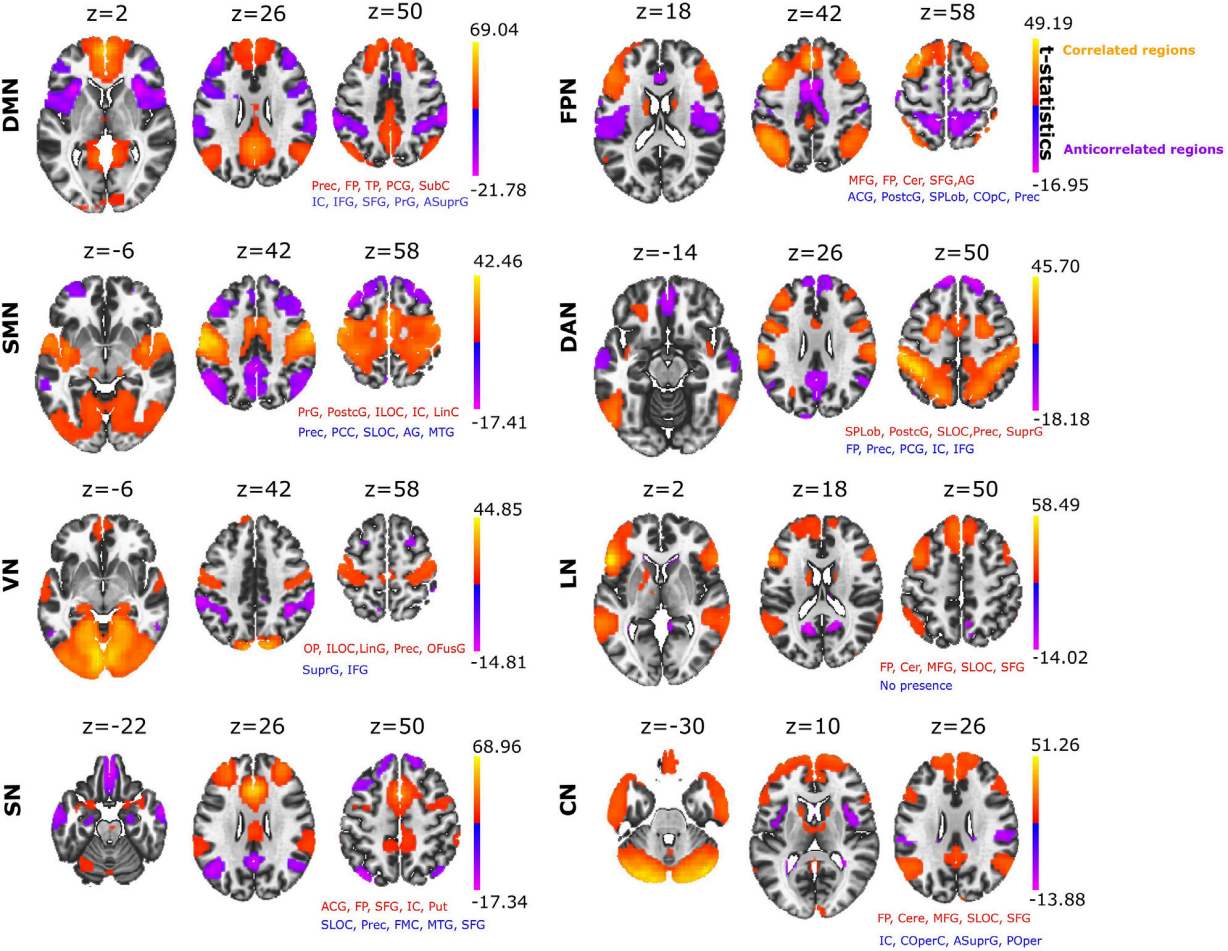
Seed based correlation analysis provides major resting state networks. Positive correlated networks (C > 0; yellow-orange) and anticorrelated networks (C < 0; blue-purple) for each of the eight networks: DMN, Default Mode Network; FPN, Fronto Parietal Network, SMN, SensoriMotor Network; DAN, Dorsal Attention Network; VN, Visual Network; LN, Language Network; SN, Salience Network; CN, Cerebellar Network. For each of the networks, the text in red and blue indicates the names of the brain structures appearing in both the positive and negative correlated networks, respectively (the name of the structure appears if the brain map overlaps with it by at least 10% or more). Abbreviations: Prec, Precuneus; FP, Frontal Pole; TP, Temporal Pole; PCG, Posterior Cingulate Gyrus; SubC, Subcallosal Cortex; IC, Insular Cortex; IFG, Inferior Frontal Gyrus; SFG, Superior Frontal Gyrus; PrG, Precentral Gyrus; ASuprG, Anterior Supramarginal Gyrus; MFG, Middle Frontal Gyrus; Cer, Cerebellum; ACG, Anterior Cingulate Gyrus; PostcG, Postcentral Gyrus; SPLob, Superior Parietal Lobule; COpC, Central Opercular Cortex; ILOC, Inferior Lateral Occipital Cortex; LinC, Lingual Cortex; SLOC, Superior Lateral Occipital Cortex; SuprG, Anterior and Posterior Supramarginal Gyrus; OP, Occipital Pole; LinG, Lingual Gyrus; OFG, Occipital Fusiform Gyrus; Put, Putamen; FMC, Frontal Medial Cortex; POper, Parietal Operculum. Voxel-level multiple correction of p-FDR-corrected < 10^^−14^ and cluster peak p-FWE corrected <10^^−14^.

SBC was used to build eight different RSNs: DMN, FPN, SMN, DAN, VN, LN, SN and CN (Figure 1). The anatomical description of each RSN is provided (Supplementary File S1). When we looked at which brain structures participated across all different ACNs (Figure 1 provides detailed voxel-level statistical t-test brain maps), we found that some structures were participating more than others. The anatomical structure with highest participation was the precuneus, which displayed significant anticorrelation in four of the eight RSNs analysed (Table 1), appearing most strongly in the SMN and reducing progressively through the DAN, SN and FPN. The next structure with the second most participation was the Anterior Supramarginal Gyrus, which was also present in the ACN of four of the eights RSNs, namely the VN, DMN, FPN and CN. The next dominant structure was the Central Opercular Cortex participating in the CAN corresponding to DMN, FPN and CN.

**TABLE 1:** Major structures fulfilling an anticorrelated role in different RSNs. The Table shows the structures most frequently present in ACN and their volumes (in brackets), measured in number of voxels with size 2 ×2×2 mm^3^. The name of the RSN with which the structures establishes an anticorrelation, and the relative degree of overlap (%) between the brain structure and each RSN is also shown.

Brain structure	Anticorrelated with	Percentage anticorrelation (%)
Precuneus (5,598)	Sensorimotor Network; Dorsal Attention Network; Salience Network; Fronto Parietal Network	30.17 16.32 11.90 9.64
Anterior Supramarginal Gyrus (1729)	Visual Network; Default Mode Network; Fronto Parietal Network; Cerebellar Network	22.90 14.34 11.91 9.48
Central Opercular Cortex (935)	Default Mode Network; Fronto Parietal Network; Cerebellar Network	57.65 56.25 16.58

**FIGURE 2:**
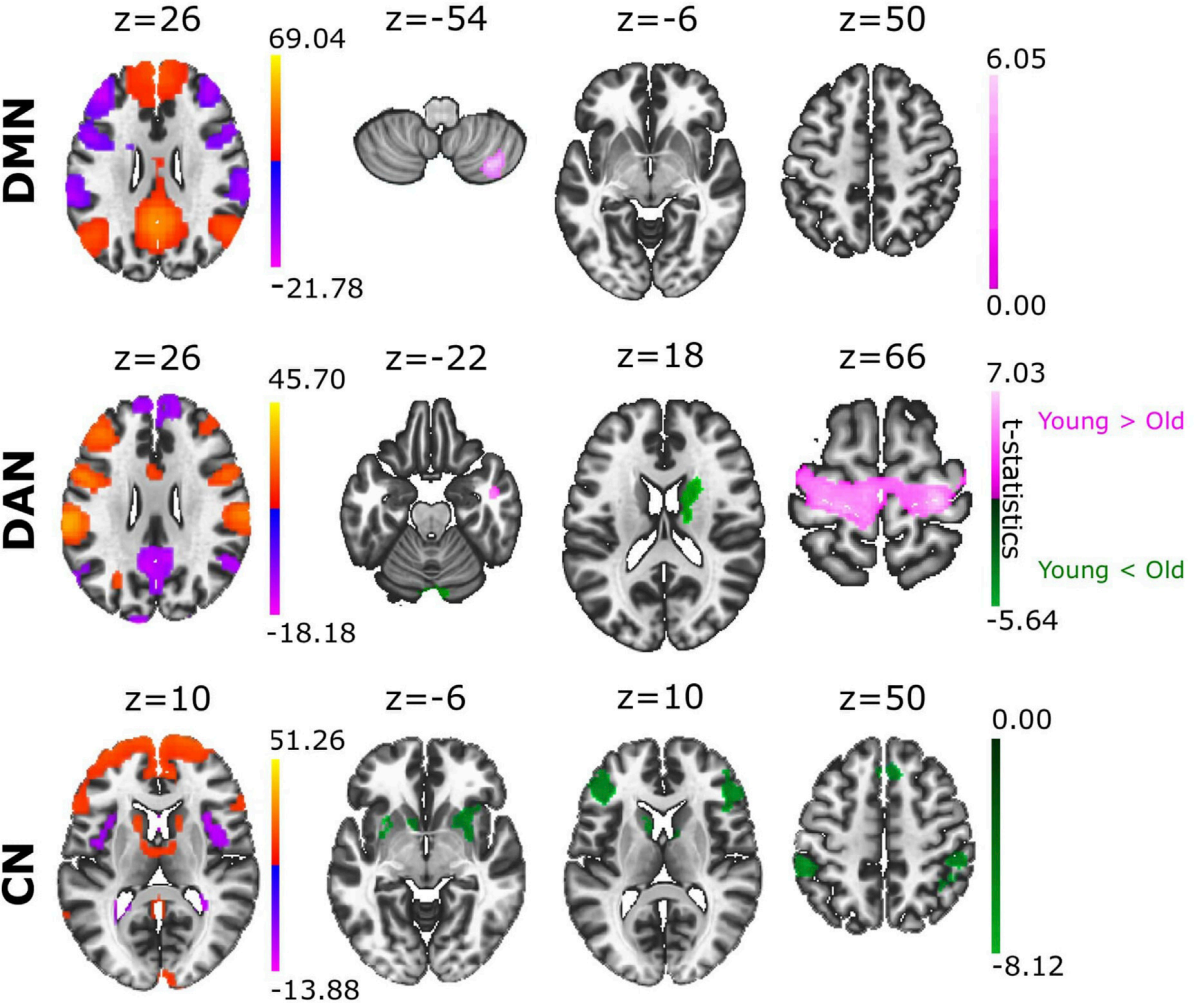
Group differences in ACN between young and old adults. Based on the contrast Young > Old (coloured in pink), the different ACN maps provided significant differences in DMN and DAN. When comparing the groups with the contrast Young < Old (coloured in green), we found significant ACN differences in DAN and CN. Voxel-level multiple correction of p-FDR-corrected < 0.05 and cluster size p-FDR-corrected < 0.05.

In relation to our hypothesis that the structure of an ACN can differentiate between brain conditions, we asked if ACN could reveal novel aspects in aging. To achieve this goal, we analysed the ACN of the eight different RSNs obtained from a sample of young and old participants (Figure 2, providing statistical t-test brain maps resulting from the group differences). Notably, we found that cerebellar regions showed greater anticorrelation with multiple brain areas in older adults than in younger adults, and this occurred in four of the eight ACNs analysed. Specifically, stronger cerebellar anticorrelations were evident in the older population in SN, DAN, FPN, and LN (Supplementary File S2). We also found a greater anticorrelation in DMN with cerebellar regions in young adults, in contrast to what was observed in the rest of the networks, namely, that the cerebellum had a stronger anticorrelated role in the older individuals. Moreover, we also found that precentral gyrus had significantly stronger anticorrelations with DAN in the young as opposed to the older participants, although the opposite contribution was found for caudate and cerebellum, as they were more strongly anticorrelated with DAN in older participants than in younger participants. When comparing ACN from CN, we found the following brain structures participating more in older participants: precentral gyrus, frontal pole, putamen and supramarginal gyrus (for details see Supplementary File S3).

To assess the association between ACN and cognitive scores, we made use of N = 1,000 subjects of the human connectome dataset (Table 2). As a control for this analysis, we also investigated the association of the same scores (total and early cognition) with the positive correlated networks (PCNs). For PCN, the size of the significantly associated clusters was (in average) larger than that for ACN. In particular, PCN provided significantly larger clusters for DMN, SMN and DAN as compared to those for ACN. However, FPN, VN, LN, SN and CN provided similar cluster size for both PCN and ACN. Of note, for the Early Childhood composite score and CN network, ACN provided had bigger clusters than PNC.

**TABLE 2:** Cognitive relevance of ACN. The table shows for each RSN (first column), the results of the voxel-level association of a given cognitive score (second column) with both PCN (third column) and ACN (fourth column). In particular, for each addressed association (a row in the table), results are given by the number of significant clusters (#Clusters), encompassing all voxels with statistically significant association, and the average size of those clusters measured in mm^3^.

RSN	Cognitive score	PCN		ACN	
		#Clusters	Average size (mm^3^)	#Clusters	Average size (mm^3^)
DMN	Early	5	993,78	15	53,35
DMN	Total	4	783,41	10	42,16
FPN	Early	18	72,16	12	59,59
FPN	Total	16	82,20	14	42,60
SMN	Early	9	193,29	4	83,75
SMN	Total	8	228,59	9	49,69
DAN	Early	6	527,46	2	42,56
DAN	Total	12	198,71	8	47,03
VN	Early	9	63,07	2	45,06
VN	Total	1	51,75	1	40,50
LN	Early	15	105,46	27	47,93
LN	Total	19	103,77	25	69,74
SN	Early	13	64,28	6	51,69
SN	Total	8	70,50	4	56,31
CN	Early	7	67,54	4	98,22
CN	Total	9	73,96	9	56,03

Abbreviations: RSN, resting state network; PCN, positive correlated networks; ACN, anticorrelated networks, a.k.a. negative correlated networks; Early, Early Childhood Composite; Total, Total Cognitive Function Composite. Only clusters larger than 50 voxels are considered in this analysis.

## Discussion

Most studies of functional connectivity at rest have analysed positive correlation networks, meaning that ACN have to some extent been neglected. Here, we describe the complete structure of ACN, obtaining a different network for each of the well-known RSNs analysed. Our data show that the spatial distribution of anticorrelation structures is very heterogeneous, and encompasses brain structures throughout the entire brain, with the strongest anticorrelation contribution found for precuneus, anterior supramarginal gyrus and central opercular cortex.

The anticorrelating role of the precuneus as part of the DMN has been proposed previously, as DMN anticorrelates with the activation of multiple regions for tasks that demand attention and mental control (Fransson, 2005; Kelly et al., 2008; Uddin et al., 2009; Crosson, 2013; Raichle, 2015; Sormaz et al., 2018). The connectivity of the precuneus is extensive and widespread, involving cortical and subcortical structures that participate in the processing of highly integrated and associative information, rather than direct processing of external stimuli (Cavanna and Trimble, 2006). Furthermore, the precuneus is functionally specialized for spatially guided behavioural processing (Selemon and Goldman-Rakic, 1988) and the activation of the precuneus precedes the onset of imagined movement, indicating that precuneus may be involved in generating spatial information related to imagined peripheral and body movements (Ogiso et al., 2000). Other studies have shown deactivation of the precuneus can be driven by different visual tasks, including visual attention (Ossandón et al., 2011) and perception (González-García et al., 2018), visual working memory and episodic memory (Sormaz et al., 2018). In psychiatric disorders, such as depressive disorders, it was also shown the anticorrelated role of the precuneus with the ventrolateral Prefrontal Cortex (Aryutova et al., 2021). This deactivation would be consistent with our findings, highlighting the critical role of the precuneus as the main anticorrelated hub across human brain networks.

The second structure with an important anticorrelation role was the anterior supramarginal gyrus, which overlaps with the SN. The supramarginal gyrus has been shown to play a key role in eliciting affective, self-other distinctions and empathic responses (Bukowski et al., 2020). Anticorrelation between SN and DMN has also been shown (Gopinath et al., 2015), in part due to the fact that SN is involved in attention demands for tasks that require cognitive control and where the insula reflects the main *core* for its implementation (Dosenbach et al., 2006). As part of the vestibular system, the supramarginal gyrus is also involved in anticorrelation with VN, integrating multisensory signals and playing an important role in visual integration (Dieterich and Brandt, 2018). In addition, we found anticorrelation for the supramarginal gyrus within FPN (also known as the Central Executive Network), consistent with a Granger causality analysis showing that SN mediates the switch between DMN and FPN (Uddin, 2015). Our findings also reveal anticorrelation from the supramarginal gyrus within CN, in agreement with previous studies relating the vestibular system and cerebellum (Hilber et al., 2019), with the cerebellum being crucial for the development of an internal model of action (Hétu et al., 2013), and the vestibular system relevant for perception, navigation and motor decision-making. Finally, the central opercular cortex is the third most relevant anticorrelation structure. Located in the Cingulo-Opercular Network (CON), it also represents a part of SN (Sestieri et al., 2011), and it is activated during the performance of some tasks like trial initiation and target detection, while it is also widely involved in a broad range of cognitive processes (Dosenbach et al., 2006).

In the second part of our work, we compared in relation to ACN two different conditions of young and old participants. Previous studies showed a decrease in the intra-network functional connectivity and increased functional inter-network connectivity in older as opposed to younger adults (Chan et al., 2014; Damoiseaux, 2017). Furthermore, decreased activation of DMN has been seen in older adults (Damoiseaux, 2017), as well as weaker anticorrelated interactions between DMN and DAN in older as opposed to younger adults (Spreng et al., 2016). Our results show higher anticorrelation in DMN of younger adults occurring in cerebellum lobule VII, which corresponds with cognitive demand functions (Stoodley et al., 2012), and with cerebellum lobule VIII that is involved in sensorimotor tasks (Stoodley et al., 2012; Fernandez-Iriondo et al., 2021). In relation to the cerebellar participation in DAN, older adults also showed stronger anticorrelation in cerebellum crus I, cerebellum crus II (corresponding to sensorimotor tasks) and the vermis VII that is involved in the proprioception of the body (Stoodley et al., 2010). We also showed stronger caudate anticorrelations in DAN for older adults, which is known to be implicated in motor processing (Nestler et al., 2020).

In the third part of this work, we have performed an analysis to measure the cognitive-relevance of ACN using N = 1,000 subjects of the human connectome dataset. For this aim, we have used two different cognitive scores, Total Cognitive Function Composite and Early Childhood Composite. While it is true that, in general, the significant (average) cluster size for positive correlated networks is larger than that of ACN (indicating a higher significant cognitive association for the former compared to the latter), however, this was not the case for all RSNs. In particular, the clusters in the positive correlated network of DMN, SMN and DAN were higher than those in ACN. However, for other networks such as FPN, VN, LN, SN and CN, a similar amount of significant association with cognitive performance (measured by the average size of clusters of significant voxels) were found for both positively correlated and anticorrelated networks. Therefore, it can be concluded that ACN have an important role in the underlying of cognitive performance. Additional studies in clinical populations, for example, in psychiatric or behavioral disorders, would be very interesting to be addressed by assessing ACN.

An important ACN role has been found for CN. In particular, the findings of stronger CN anticorrelations in older adults might explain some of the functional alterations that occur with age (Deng et al., 2019), particularly in several cognitive functions like movement control, executive coordination or emotional regulation (Fernandez-Iriondo et al., 2021) but also, it might be related to changes in cerebellar morphometric volume (Han et al., 2020). In line with these results, our analysis of cognitive association with ACN provided higher clusters for CN.

To summarize, our results have shed some light into the structure and organization of ACNs, have revealed that their study may be useful to approach aging, and this is extensible to other pathologies, and furthermore, that ACN has a significant association with cognitive performance. In the same way that multiple studies have addressed the activation relation between task-fMRI and resting networks for positively correlated networks (Smith et al., 2009; Di et al., 2013; Cole et al., 2014; Rasero et al., 2018), future complementary studies might also address the relation of ACN in task-specific fMRI as compared to rest.

## Data Availability

Publicly available datasets were analysed in this study. This data can be found here: http://fcon_1000.projects.nitrc.org/fcpClassic/FcpTable.html, http://fcon_1000.projects.nitrc.org/indi/retro/MPI_LEMON.html, https://www.humanconnectome.org.

## Supplementary material

Supplementary File S1 Eight RSNs with positive correlated and anticorrelated structures (considered if their volume overlapped with the networks at least 10% or more) obtained from the Beijing dataset. The seed chosen for each network is indicated; the positive correlated structures are shown in red while the anticorrelated are in blue. Furthermore, the spatial localization of cluster centroid, the cluster size and the used statistics are described for each cluster.

Supplementary File S2 A table showing the description of each structure (in number of significant voxels) involved in positive correlated and anticorrelated networks for each RSN according to the different used seeds and obtained from the Beijing dataset. In the case in which the structure is bilateralised (left and right), we report the average size between both hemispheres. The seed chosen is also shown, the positive correlated structures are shown in red, and the anticorrelated structures in blue.

Supplementary File S3 The anticorrelated clusters for each of the eight different RSNs obtained from the LEMON dataset are described. The seed chosen for each network is shown, the pink text refers to significant structures resulting from the contrast Young > Old, while the green text represents the opposite contrast. The spatial localization of cluster centroid, the cluster size and the different statistics measured are described for each cluster.
